# Efficiency and consistency enhancement for alkaline electrolyzers driven by renewable energy sources

**DOI:** 10.1038/s44172-023-00070-7

**Published:** 2023-05-03

**Authors:** Yanghong Xia, Haoran Cheng, Hanghang He, Wei Wei

**Affiliations:** College of Electrical Engineering, Zhejiang University, 310027 Hangzhou, Zhejiang Province China

**Keywords:** Hydrogen energy, Energy conservation, Electrical and electronic engineering

## Abstract

Low-cost alkaline water electrolysis from renewable energy sources (RESs) is suitable for large-scale hydrogen production. However, fluctuating RESs lead to poor performance of alkaline water electrolyzers (AWEs) at low loads. Here we explore two urgent performance issues: inefficiency and inconsistency. Through detailed operation process analysis of AWEs and the established equivalent electrical model, we reveal the mechanisms of inefficiency and inconsistency of low-load AWEs are related to the physical structure and electrical characteristics. Furthermore, we propose a multi-mode self-optimization electrolysis converting strategy to improve the efficiency and consistency of AWEs. In particular, compared to a conventional dc power supply, we demonstrate using a lab-scale and large-scale commercially available AWE that the maximum efficiency can be doubled while the operation range of the electrolyzer can be extended from 30–100% to 10–100% of rated load. Our method can be easily generalized and can facilitate hydrogen production from RESs.

## Abstract (web summary)

Renewable energy sources could be used to drive alkaline electrolyzers for large-scale hydrogen production. Here Yanghong Xia and colleagues show that the fluctuating nature of renewable energy sources compromises the efficiency and consistency of electrolyzer performance. They also show that a strategy of multi-mode self-optimization can address this problem, extending the range of operation as well as improving efficiency at low loads.

## Introduction

Nowadays, hydrogen has gotten great attention because of the pronounced environmental and climate problems caused by carbon-intensive fossil energies^1,2^. With its clean, versatile, and lightweight properties, hydrogen is considered the most promising solution that can help reduce the carbon emissions of transportation^3^, metallurgy^4^, chemical industry^5^, and other sectors^6^. As a result, the hydrogen demand has been growing exponentially, reaching 70 million tons in 2018 and is expected to reach 545 million tons per year in 2050^7^. However, most of the world’s hydrogen is currently obtained by the reformation of fossil energies, which consumes much energy and causes global CO_2_ emissions to reach more than 830 million tonnes per year^7^. For sustainable development, hydrogen production must be efficient and environmentally friendly. Therefore, hydrogen production technology by electrolyzing water using surplus photovoltaic power, wind power, and other renewable electricity, namely green hydrogen, has become a hot research topic^8,9^.

Currently, there are three electrolytic hydrogen methods, solid oxide electrolyzers (SOEs), proton exchange membrane (PEM) electrolyzers, and AWEs. SOEs constitute an advanced concept enabling water or steam electrolysis at high temperatures (600–900 °C)^10^, whose efficiency is higher than PEM electrolyzers and AWEs. As for the practical application, SOEs meet remarkable challenges as concerns the thermal stability of materials, gas mixture, and sealing issues. Hence, SOEs are still at the R&D stage. Compared to SOEs, PEM electrolyzers and AWEs are commercially available. PEM electrolyzers are more efficient and allow for higher current densities than AWEs. One obvious disadvantage of PEM electrolyzers is the high capital cost of their acid-tolerance components like the Nafion membrane, titanium bipolar plates, and novel metal catalysts PT/C and IrO_2_^11^. In addition, their shorter lifetimes than AWEs have also hindered their application in large-scale power-to-gas scenarios^12^. In contrast, AWEs is a relatively mature technology that has been developed over 100 years. For commercial AWEs, earth-abundant electrocatalysts are stable enough to run both half-reactions, whose lifetime can reach up to 15 years. Hence, AWEs are very suitable for large-scale electrolytic hydrogen projects^13,14^.

Although projects with AWEs up to 6 MW exist in practice^15^, the operational flexibility of AWEs still needs to be improved, especially when they are powered by wide-range fluctuant RESs. One widely concerned challenge is the impurity problem that the low-load AWEs (usually 25–45% of rated load) could potentially lead to gas crossover between the cathode and anode. This impurity will result in the formation of flammable gas mixture^16^, especially for the anode where 2 vol% H_2_ in O_2_ corresponds to about 50% of the lower explosive limit. Therefore, when the supplied RESs, like photovoltaic power, frequently fluctuate in a wide range, the start-stops of AWEs are increased obviously to ensure the system’s safety. These frequent start-stops have a great influence on the stability and power quality of the electrical power system^17,18^; at the same time, the RESs cannot be fully consumed because of the low-load curtailment of AWEs. In addition, the long-term shutdown will cause reverse currents for the AWEs^19–21^, which will adversely affect the durability of electrodes. Steady and dynamic models of gas impurity caused by gas crossover are established, considering several influence factors. To sum up, the gas impurity problem is caused by two reasons: the crossover through the diaphragm by gas diffusion^22,23^ and the crossover by the circulated electrolyte mixing^24,25^. To enhance the gas purity for low-load AWEs, several strategies are also proposed. Anion exchange membrane^26,27^ and other novel diaphragm structures are developed to prevent the crossover through the diaphragm by gas diffusion. Based on the same goal, Qi et al. propose a pressure control strategy to extend the load range of AWEs^28^. On the other hand, Schug regulates the electrolyte circulation rate adaptively to reduce the crossover by the circulated electrolyte mixing^29^. In order to solve the gas impurity problem completely, a novel alkaline electrolysis system is designed^30^, which separates hydrogen and oxygen evolution. But its reliability needs to be further verified.

Through the above strategies, the impurity problem of low-load AWEs has been alleviated. However, in this work, we find that the inefficiency and inconsistency problems still hinder the operation of low-load AWEs, except for the impurity problem. For the low-load AWEs, the efficiency of electrolyzers is very low, and the operation states of different cells of electrolyzers are not consistent. The inefficiency problem will cause extra energy dissipation, and the inconsistency problem will cause obvious lifetime degradation of long-running cells. Because corresponding mechanisms are not analyzed effectively, some conventional methods, like novel catalysts^31^, highly consistent cells of AWEs^32^, and so on, cannot well solve these two problems. Here, we analyze the detailed operation process of AWEs and establish the equivalent electrical model. Then, the inefficiency and inconsistency mechanisms of low-load AWEs are revealed. It is found that the physical structures and electrical characteristics but not the chemical properties of AWEs have a decisive influence on the low-load performance. Based on this, a multi-mode self-optimization electrolysis converting strategy is proposed to enhance the efficiency and consistency of AWEs. Its effectiveness is verified by a 2 Nm^3^/h commercial AWE, except for the lab-scale test system. Especially compared to the conventional dc power supply, the electrolyzer efficiency is increased from 29.27 to 53.21% under 15% of the rated load for the 2 Nm^3^/h commercial AWE. At the same time, under the condition that the system efficiency is larger than 50%, the operation range of the electrolyzer is extended from 30~100% to 10~100% of the rated load. The proposed method just changes the power supply and does not need to modify the components of electrolyzers; it can be easily generalized and can facilitate hydrogen production from RESs.

## Results

### Problem presentation

To illustrate the inefficiency and inconsistency phenomena of low-load AWEs, several experiments are conducted based on a 2 Nm^3^/h at 80 °C commercial AWE (electrical power is about 10 kW at 80 °C) and a simple AWE. The 2 Nm^3^/h commercial AWE is mainly used for practical verification, while the simple AWE is mainly designed for principal experiments due to the convenient observation and measurements.

**Fig. 1:**
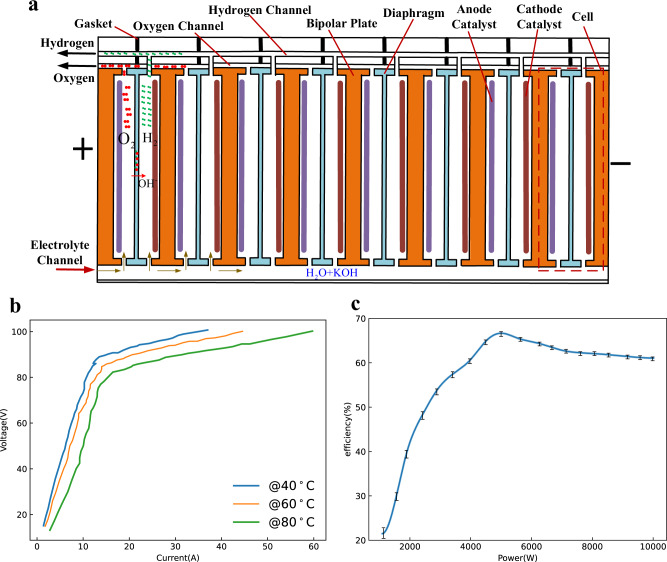
Low-load experiments of the commercial AWE. These experiments can illustrate the inefficiency phenomenon. The whole AWE consists of 48 cells, and the maximum current density is 600 mA/cm^2^; the electrolyte is an aqueous solution of KOH at 30 wt.%. The whole system is supplied by the dc voltage source. The electrical power of the AWE is about 10 kW at 80 °C. **a** Principle structure of commercial AWEs (bipolar form). **b** Voltage-current curves under different temperatures; here, the voltage is the terminal voltage of the whole electrolyzer, and the current is the input current of the whole electrolyzer. The experiment was repeated five times. **c** Efficiency-power curves at 80 °C; here, the power is the input power of the whole electrolyzer, and efficiency = HHV of H_2_/ Electric energy consumption. Error bars in (**c**) denote the standard deviation where $n=5$. The experiment was repeated five times. The source data for Fig. 1b,c are available in Dataset 1.

As shown in Fig. 1a, the cell is the basic element of the AWE; the cell consists of bipolar plates, anode/cathode catalysts, and a diaphragm. Usually, commercial AWEs are the bipolar form^14,25,29,33^, composed of several cells connected in series (for the adopted AWE, the total cells are 48). Under this condition, each electrode, except for the first and the last ones, has two polarities, the anode side and the cathode side, which belong to two adjacent cells. In addition, the hydrogen channel, oxygen channel, and electrolyte channel are shared by all cells. For the adopted AWE, the electrolyte is an aqueous solution of KOH at 30 wt.% since its ionic conductivity is the highest at this concentration.

When the AWE is powered by the dc voltage source from 0 to 100 V, the relationship between the total electrolytic voltage and electrolytic current is presented in Fig. 1b. From the figure, the following observations should be noticed. (1) According to the series circuit, the theoretical reserve voltage of the whole electrolyzer is about 59 V (48$\times$1.23 V). However, under 59 V, the electrolytic current is still generated. (2) For the low-load range (<15 A) and high-load range (>15 A), there is an obvious linear relationship between the voltage and current, but with different slopes, the low-load equivalent resistance is much larger than the high-load equivalent resistance. That is, the system parameters or states are changed. (3) In the whole range, the relationship between the electrolytic voltage and electrolytic current does not meet the typical electrolysis hydrogen model presented in refs. ^14,33,34^.

Fig. 1c shows the system efficiency curves with the variation of input power. Here, efficiency is defined as

1 $$\eta =\frac{{{{{{\rm{HHV}}}}}}\,{{{{{\rm{of}}}}}}\,{{{{{{\rm{H}}}}}}}_{2}}{{{{{{\rm{Electric}}}}}}\,{{{{{\rm{energy}}}}}}\,{{{{{\rm{consumption}}}}}}}$$

For the single cell, Eq. (1) can be further expressed as

2 $$\eta ={\eta }_{F}\frac{HH{V}_{H2}\cdot {\int }_{{t}_{1}}^{{t}_{2}}{{I}_{cell}}^{dt}}{2F{\int }_{{t}_{1}}^{{t}_{2}}{V}_{cell}\cdot {{I}_{cell}}^{dt}},$$

where ${\eta }_{F}$ is the Faraday efficiency, $F$ is the Faraday constant, ${{{{{{\rm{HHV}}}}}}}_{{{{{{\rm{H}}}}}}2}$ is the higher heating value of 1 mol hydrogen, ${I}_{{cell}}$ is the electrolytic current, and ${V}_{{cell}}$ is the electrolytic voltage.

Based on Eq. (2), if the electrolyzer is considered the pure series of multiple cells, the system efficiency is decreased as the input power is increased when it is supplied by the dc voltage source. However, From Fig. 1c, it can be seen that the system efficiency changes non-monotonically with the variation of input power. The low-load efficiency is lower than the high-load efficiency, obviously. Especially the system efficiency is lower than 30% under 15% of the rated load. These experimental results illustrate the inefficiency phenomenon of low-load AWEs.

**Fig. 2:**
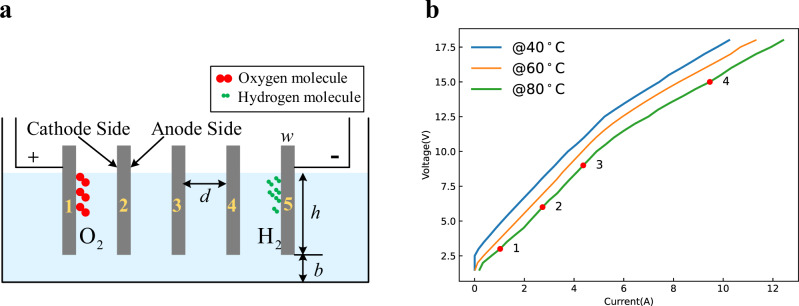
Low-load experiments of the simple AWE. These experiments can illustrate the inconsistency phenomenon. The small-scale AWE simulates the large-scale commercial AWE. The bottom channel (the height is *b*) simulates the electrolyte channel, and there are four cells. Its bipolar plates are stainless steel; the electrolyte is an aqueous solution of KOH at 30 wt.%. The whole system is supplied by the dc voltage source. **a** Principle structure of the simple AWE, the size of the electrolyzer is 50 × 10 × 12 cm, the height of bottom channel *b* = 0.2 cm, the height of bipolar plates *h* = 6 cm, the size of bipolar plates is 10 × 10 cm and the width *w* = 0.1 cm, the distance between bipolar plates *d* = 12 cm. **b** Voltage-current curves at different temperatures; here the voltage is the terminal voltage of the whole electrolyzer, and the current is the input current of the whole electrolyzer. The experiment was repeated five times. The source data for Fig. 2b are available in Dataset 1.

To further know the operation process of the AWE, the low-load experiments of a simple AWE are conducted for its convenient observation and measurements. As shown in Fig. 2a, except for the electrode materials, the small-scale AWE has a similar structure to the large-scale commercial AWE.

**Fig. 3:**
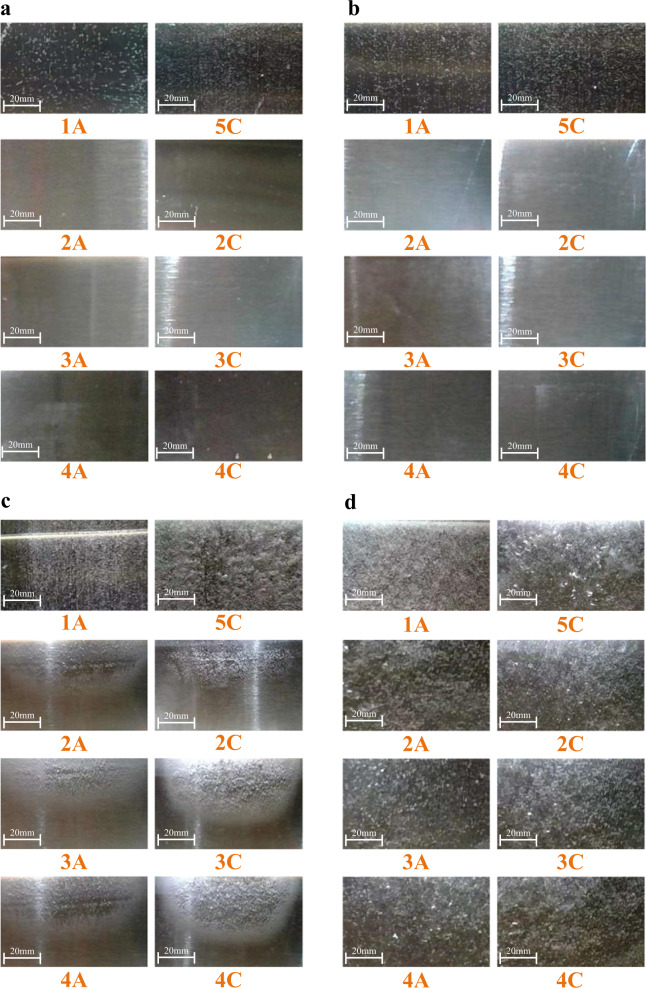
Operating states of different cells under different electrolytic voltage at 80 °C. **a** 3 V < the reserve voltage 4.92 V. **b** 6 V > the reserve voltage 4.92 V. **c** 9 V > the reserve voltage 4.92 V. **d** 15 V > the reserve voltage 4.92 V.

The relationship between the total electrolytic voltage and electrolytic current is presented in Fig. 2b, which is similar to Fig. 1b. Fig. 3 shows the operating states of different cells under the operating point 1 in Fig. 2b, where the electrolytic voltage is 3 V and is less than the reserve voltage 4.92 V (4$\times$1.23 V). It can be seen that only the first and the last bipolar plates have obvious bubbles, and the middle plates do not have any bubbles. It can verify the existence of current when the electrolytic voltage is less than the reserve voltage. At the same time, it also means that the current only flows through plate1 and plate5 but does not flow through the middle plates. That is, electrolytic reactions occur only on the solid–liquid interfaces of plate1 and plate5 but do not occur on the solid–liquid interfaces of middle plates. Therefore, the operating states of different cells of low-load AWEs are not consistent, namely, the aforementioned inefficiency phenomenon.

Increasing the electrolytic voltage to 6 V, which is larger than the reserve voltage 4.92 V, namely the operating point 2 in Fig. 2b, the operating states of different cells are shown in Fig. 3b. However, it can be seen that there are still no reactions on the middle plates, while the reactions on plate1 and plate5 are stronger. Continuously increasing the electrolytic voltage to 9 V, namely the operating point 3 in Fig. 2b, it can be seen that the upper parts of the middle plates have reactions, but the lower parts of the middle plates still have no reactions as shown in Fig. 3c. The middle plates have obvious reactions as shown in Fig. 3d till the electrolytic voltage reaches to 15 V, namely the operating point 4 in Fig. 2b, which is much larger than the reserve voltage. Hence, the electrolyzer cannot be viewed as a simple series of multiple cells, and its operation process is not just a generalization of the single cell; more is different^35^.

### Mechanisms analysis

**Fig. 4:**
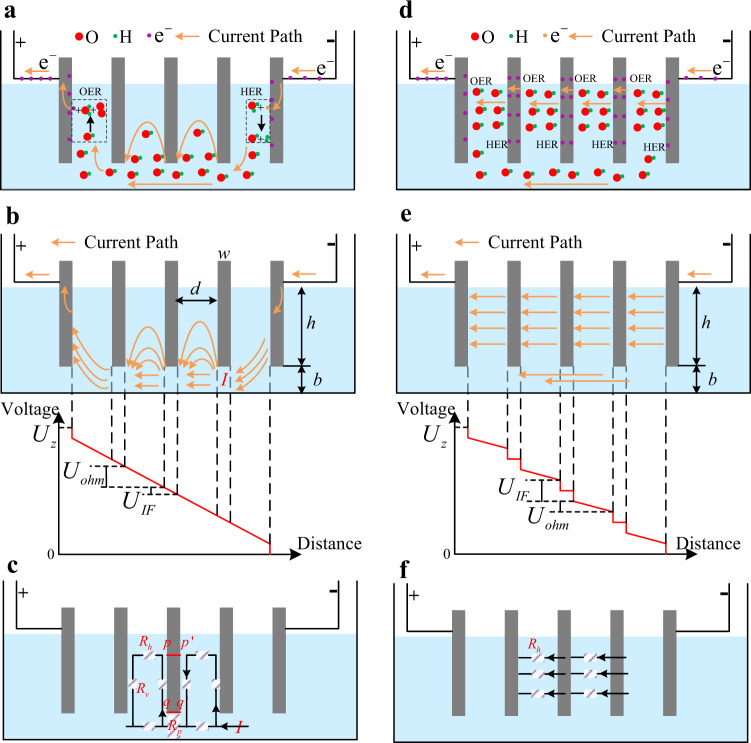
Electrochemical process of AWEs. If the bipolar plate produces electrolytic reactions, its interfacial potential difference must be larger than the reserve voltage. Electrolytic reactions include Oxygen Evolution Reaction (OER) and Hydrogen Evolution Reaction (HER). For commercial AWEs, the electrolyte channel connects the first and the last bipolar plates; they can form a cell. Hence, the electrolytic current can be generated under the low terminal voltage. However, the interfacial potential differences of the middle plates are only generated by the voltage drop of this electrolytic current through the electrolyte channel. Here, ${U}_{Z}$ is the terminal voltage of the electrolyzer, ${U}_{{IF}}$ is the interfacial potential difference, ${U}_{{ohm}}$ is the ohmic voltage, ${U}_{Z}=N\cdot({U}_{{IF}}+{U}_{{ohm}})$, $N$ is the number of cells. **a** Electrochemical process and current path of the low-load AWE. **b** Voltage changes with distance of the low**-**load AWE. **c** Low-load equivalent circuit. **d** Electrochemical process and current path of the high-load AWE. **e** Voltage changes with distance of the high-load AWE. **f** High-load equivalent circuit.

In this section, the mechanisms of the inefficiency and inconsistency of low-load AWEs are analyzed. Fig. 4a shows the electrical process of low-load AWEs. Due to the electrolyte channel, the connection of bipolar plates is not the pure series; any two of them are connected. Especially the first and the last bipolar plates are connected. Hence, when the terminal voltage of the electrolyzer ${U}_{z}$ >the reserve voltage of one cell ${U}_{o}$(namely 1.23 V), the OER occurs on the solid–liquid interfaces of the positive electrode, and the HER occurs on the solid–liquid interfaces of the negative electrode. The hydroxide ions pass through the electrolyte channel and partial cell spaces, as shown in Fig. 4a, then the electrolytic current is generated, which is called the start-up current in this paper. This can explain the experimental results shown in Fig. 3a.

The interfacial potential differences of the middle plates are generated by the voltage drop of the start-up current. From the current paths in Fig. 4b and the equivalent circuit in Fig. 4c, it can be seen that the interfacial potential differences between the upper parts of the middle plates are larger than that of the lower parts of the middle plates. For example, as shown in the figure, the interfacial potential differences ${U}_{{\!\!pp}\prime}$ between points *p* and *p’* is larger than the interfacial potential differences ${U}_{{\!\!qq}\prime}$ between points *q* and *q’*. Hence, with the increase of the start-up current *I*, the upper parts of the middle plates produce electrolytic reactions more easily than the lower parts of the middle plates. This can explain the experimental results shown in Fig. 3c.

Furthermore, as shown in Fig. 4b,c, considering the ohmic voltage drop between two plates ${U}_{{\!ohm}}$, the terminal voltage can be expressed as

3 $${U}_{z}=N\cdot ({U}_{IF}+{U}_{ohm}),$$

where $N$ is the number of cells. From the figures, it can be seen that ${R}_{P}$ is proportional to the width of bipolar plates *w*. So, ${U}_{{IF}}$ is proportional to *w* too. ${U}_{{\!\!ohm}}$ is proportional to the distance between bipolar plates *d*. For the electrolyzer with large $d/w$, ${U}_{z} > N\cdot{U}_{o}$ does not mean that ${U}_{{IF}}\, > \,{U}_{o}$. That is, when ${U}_{z}\gg N\cdot{U}_{o}$ or $I$ is very large, the middle plates produce electrolytic reactions. This can explain the experimental results shown in Fig. 3b,d.

Through the above analyses, the inconsistency mechanism of low-load AWEs is revealed. In the following part, the inefficiency mechanism of low-load AWEs will be explored.

With the increasing of *I*, ${U}_{{IF}}$ will be larger than ${U}_{o}$, then the middle plates produce electrolytic reactions as shown in Fig. 4d. The hydroxide ions mainly pass through the cell spaces; the corresponding current paths and equivalent circuit are shown in Fig. 4e, f. Usually, the height of bipolar plates *h* (corresponding to the cross-sectional area) is much larger than the height of electrolyte channel *b* (corresponding to the cross-sectional area), the resistance of the current following through cell spaces is much smaller than the resistance of the current following through the electrolyte channel. At the same time, under the high-load condition, the vertical current paths are not necessary, which further reduces that resistance. In conclusion, the whole equivalent high-load resistance is much smaller than the whole equivalent low-load resistance. Therefore, the low-load efficiency of AWEs is much lower than the high-load efficiency of AWEs. This can explain the experimental results shown in Figs. 1b, c and  2b.

**Fig. 5:**
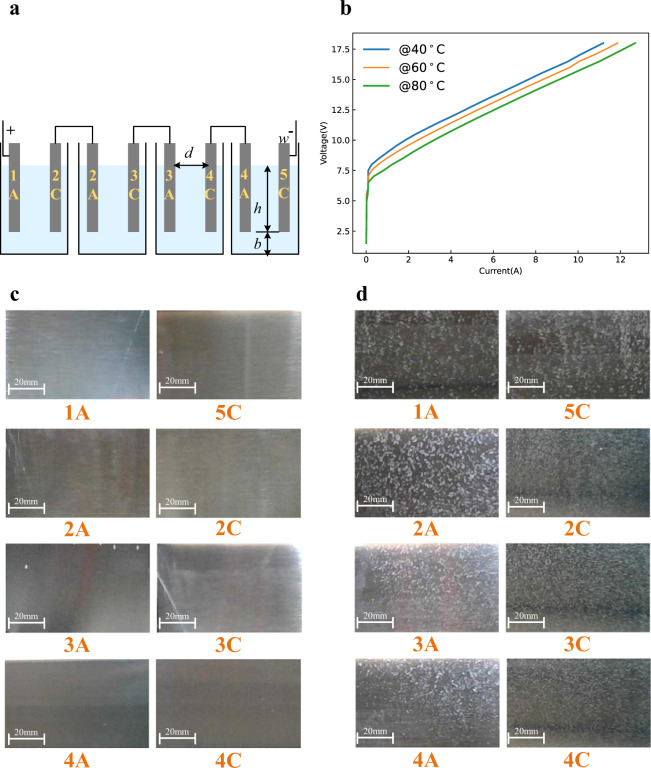
Confirmation experiment 1. Cells are connected in pure series. **a** Principle structure. **b** Voltage-current curves at different temperatures; here, the voltage is the terminal voltage of the whole electrolyzer, and the current is the input current of the whole electrolyzer. The experiment was repeated five times. The source data for Fig. 5b are available in Dataset 1. Operating states of different cells under different electrolytic voltage at 80 °C. **c** 3 V < the reserve voltage 4.92 V. **d** 9 V > the reserve voltage 4.92 V.

To verify the proposed principle, more confirmation experiments are conducted. First, the AWE consisting of purely serial cells, as shown in Fig. 5a, is studied. Except that different cells are separated and are connected in pure series, other parameters are kept the same as in Fig. 2. For the purely serial cells, since there is no electrolyte channel that connects all bipolar plates, there is no electrolytic current when the terminal voltage is lower than the reserve voltage as shown in Fig. 5b, c. At the same time, the interfacial potential differences of bipolar plates are the capacitor voltages of the electrical double layers, which share the terminal voltage equally. Therefore, when the terminal voltage is larger than the reserve voltage, all plates are conducted simultaneously, as shown in Fig. 5d. Furthermore, the voltage that makes all plates generate reactions is much lower. This conclusion can be verified through the comparison between Figs. 2b and 5b. These comparisons can further illustrate the widely adopted bipolar AWE structure shown in Fig. 1a cannot be regarded as a simple series of different cells.

**Fig. 6:**
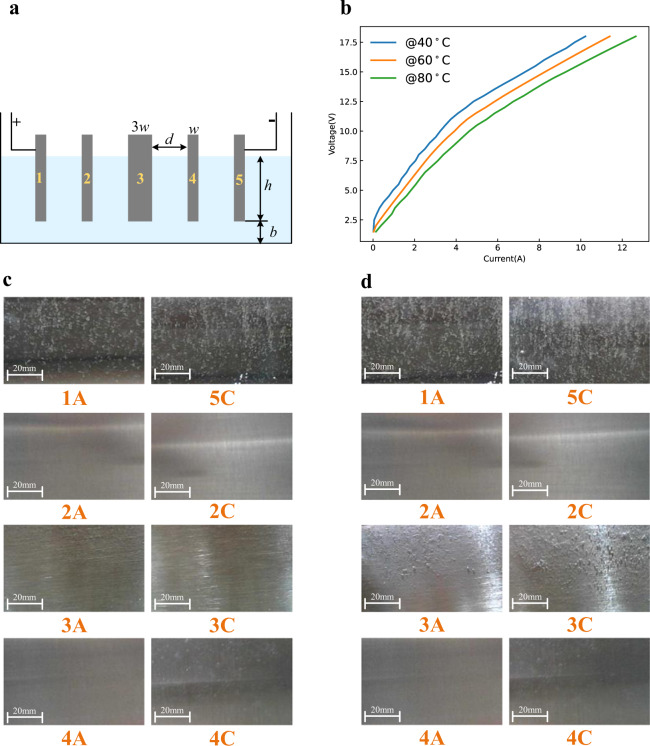
Confirmation experiment 2. Bipolar plates with different widths, here, the width of plate3 is three times that of other plates. **a** Principle structure. **b** Voltage-current curves at different temperatures; here, the voltage is the terminal voltage of the whole electrolyzer, and the current is the input current of the whole electrolyzer. The experiment was repeated five times. The source data for Fig. 6b are available in Dataset 1. Operating states of different cells under different electrolytic voltage at 80 °C. **c** 3 V < the reserve voltage 4.92 V. **d** 7 V > the reserve voltage 4.92 V.

In addition, bipolar plates with different widths are studied, as shown in Fig. 6a, where the width of plate3 is three times that of other plates. From the former analysis, for the bipolar electrolyzer with a shared electrolyte channel, the interfacial potential differences of the middle plates are generated by the voltage drop of the start-up current. Hence, for the wider bipolar plates, their interfacial potential differences are larger than the narrower ones. With the increase of the start-up current, the first and the last bipolar plates are first conducted, then the wider bipolar plates are conducted, and finally, the narrower ones are conducted, as shown in Fig. 6c, d; the whole operation process is divided into several states as shown in Fig. 6b.

### Solutions

**Fig. 7:**
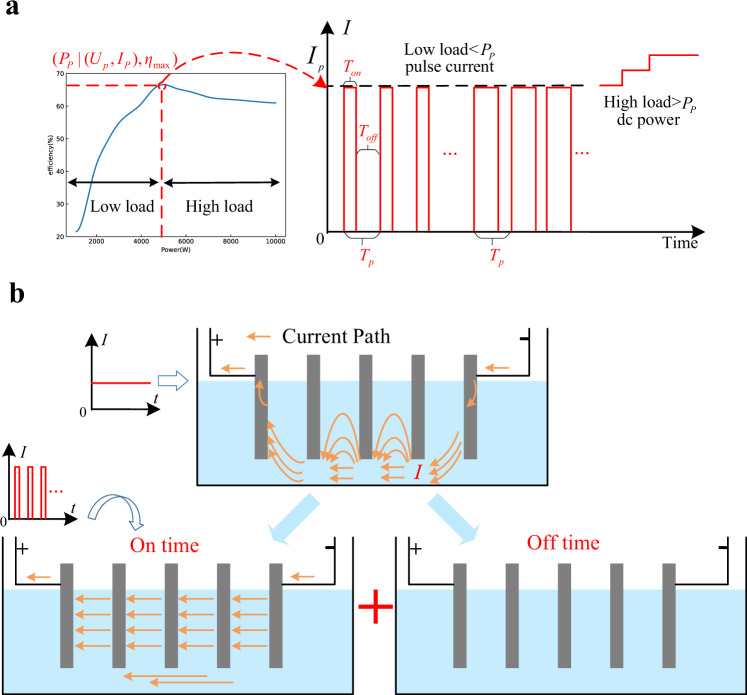
Multi-mode self-optimization electrolysis converting (MMSOEC) strategy. **a** Principle of MMSOEC strategy.$\,{T}_{{on}}$ represents on time,$\,{T}_{{off}}$ represents off time, ${T}_{p}$ represents cycle time.$\,{P}_{p}$ is the optimal system power, ${U}_{p}$ and ${I}_{p}$ are the optimal working voltage and current. ${\eta }_{{{\max }}}$ is the optimal efficiency. **b** Mechanism explanation about why MMSOEC strategy can enhance efficiency and consistency.

The inefficiency and inconsistency mechanisms of low-load AWEs have been revealed in the former section; the shared electrolyte channel and relatively larger equivalent low-load resistance are the main reasons. One intuitive idea is to make the low-load operating conditions similar to the high-load operating conditions. Based on this thought, a multi-mode self-optimization electrolysis converting (MMSOEC) strategy is proposed, as shown in Fig. 7a. Being different from the conventional dc power supply, the MMSOEC strategy provides pulse current for AWEs during the low-load period, while keeps the dc power supply for AWEs during the high-load period. The switching between low-load and high-load control is adaptive according to the power command. Especially for the low-load state, the AWEs are on and off alternately with the large current magnitude. Since the subsequent links, like the counterbalance valve, purification, storage, and so on, can be viewed as inertial links, being similar to the pulse-width modulation (PWM) circuits^36,37^, the pulsed gas does not influence the final production.

Since the AWEs have large electrical double-layer capacitors, pulse voltages will cause great non-Faradic currents. This great current will damage the power supply circuits. Hence, pulse currents instead of pulse voltages are chosen as the implementation measures for the low-load state. By changing the pulse width or duty ratio (namely ${T}_{{on}}/\,{T}_{P}$), the MMSOEC strategy regulates the system power accurately. The magnitude of pulse current ${I}_{P}$ is the optimal working current of AWEs supplied by a dc power source, under which the efficiency of the AWEs is the highest. To decrease the charge and discharge loss of electrical double-layer capacitors, the frequency is chosen as 10 Hz (namely ${T}_{P}=0.1\,{{{{{\rm{s}}}}}}$). This pulse current can be easily realized through mature power electronics technologies.

As shown in Fig. 7a, the low-load state is defined as that the system power is lower than ${P}_{P}$ (the system power under the optimal working current ${I}_{P}$), and the high-load state is defined as that the system power is higher than ${P}_{P}$. For the low-load AWEs, during on time, the provided working current is large enough that it can conduct all bipolar plates like the high-load situation. During off time, the provided working current is zero; AWEs stop working, and only the electrical double-layer capacitor discharges. In this way, the AWEs always operate under the optimal conditions, as shown in Fig. 7b. For the high-load AWEs, the dc power supply method is still adopted, and the system power is regulated through the current magnitude. Therefore, under the MMSOEC power supply, the efficiency and consistency of low-load AWEs can be greatly enhanced.

It should be noted that for the small-scale single cell (electrical power < 100 W), the effects of high-frequency pulse electrolysis (>10 kHz) have been reported. However, since the natural mechanisms are not analyzed effectively, the related results about high-frequency pulse electrolysis are confused and cannot be unified^38^. In ref. ^39–42^, the magnitude or mean value of pulse voltages equal to the magnitude of dc voltages, it is concluded that high-frequency pulse can enhance the system efficiency from three possible aspects, namely, reactant concentration, bubble detachment, and electrical double layer. However, for the pulse power supply and dc power supply, the same magnitude or mean value of voltages does not mean the same electric power. Indeed, from the view of energy, high-frequency pulse electrolysis will introduce lots of voltage or current harmonics, which will not produce hydrogen and cause obvious efficiency loss^43–45^. For the proposed MMSOEC strategy, the motivation is totally different from that of ref. ^39–42^. The fundamental motivation is based on the macroscopic equivalent circuit; the choice of pulse parameters is well-founded and is greatly different from that of refs. ^39–42^.

**Fig. 8:**
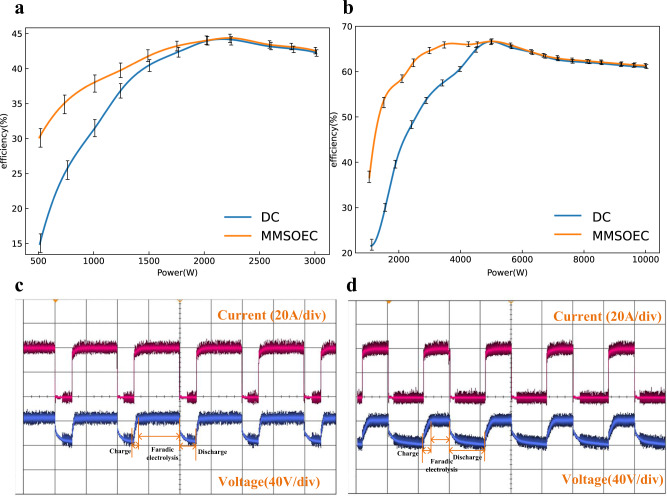
MMSOEC power supply experiments. **a** Efficiency enhancement of the lab-scale test electrolyzer at 80 °C, where ${I}_{P}=30\,{{{{{\rm{A}}}}}}$, ${T}_{P}=0.1\,{{{{{\rm{s}}}}}}$. There are 25 cells, its bipolar plates are stainless steel, and the electrolyte is an aqueous solution of KOH at 30 wt.%. The size of the electrolyzer is 41 × 10 × 12 cm, the height of bottom channel *b* = 0.5 cm, the height of bipolar plates *h* = 9 cm, the size of bipolar plates is 10 × 10 cm, and the width *w* = 0.1 cm, the distance between bipolar plates *d* = 1.6 cm. Error bars in (**a**) denote the standard deviation where $n=5$. The experiment was repeated five times. The source data for Fig. 8a are available in Dataset 1. **b** Efficiency enhancement of the commercial AWE at 80 °C, where ${I}_{P}=40\,{{{{{\rm{A}}}}}}$, ${T}_{P}=0.1\,{{{{{\rm{s}}}}}}$. Error bars in (**b**) denote the standard deviation where $n=5$. The experiment was repeated five times. The source data for Fig. 8b are available in Dataset 1. **c** Electrolytic voltage and current of the commercial AWE under the MMSOEC power supply when duty ratio = 0.75. **d** Electrolytic voltage and current of the commercial AWE under the MMSOEC power supply when duty ratio = 0.45. Here, the power is the input power of the whole electrolyzer, and efficiency = HHV of H_2_/ Electric energy consumption.

Fig. 8a shows the effects of the proposed MMSOEC strategy based on the lab-scale test electrolyzer, whose structure is similar to Fig. 2a, but the parameters are different. It is obvious that compared to the conventional dc power supply, the system’s low-load efficiency is greatly enhanced through the proposed MMSOEC strategy. Especially the efficiency is increased from 15.36 to 29.78% under 500 W, which exceeds two times. For the MMSOEC strategy, the high-load state still adopts the dc power supply; hence the system efficiency keeps the same.

To further verify the effectiveness of the proposed solutions, MMSOEC power supply experiments for the 2 Nm^3^/h commercial AWE are conducted. Fig. 8b shows system efficiency with the changes in input power under the proposed MMSOEC power supply. From the figure, it can be seen that the system’s low-load efficiency is greatly enhanced. Especially compared to the conventional dc power supply, the electrolyzer efficiency is increased from 29.27 to 53.21% under 15% of the rated load, namely 1500 W. Under the condition that the system efficiency is larger than 60% (92% of the maximum efficiency), the operation range of the electrolyzer is extended from 40~100% to 22~100% of the rated load. Furthermore, if the system efficiency is not smaller than 50% (77% of the maximum efficiency), the operation range of the electrolyzer is extended from 30~100% to 10~100% of the rated load. Results of Fig. 8a, b suggest that the MMSOEC strategy is not only the principle verification but also with great practical value; it can be generalized to related practical projects.

Fig. 8c, d show the voltage and current waveforms of the commercial AWE. It can be seen that under the low-load condition, the AWE is on and off alternately. In the on state, the electrolytic current is much larger than the electrolytic current generated by the dc power source, as shown in Fig. 1b. In addition, from the voltage waveforms, the charge and discharge processes of electrical double-layer capacitors can be clearly observed.

**Fig. 9:**
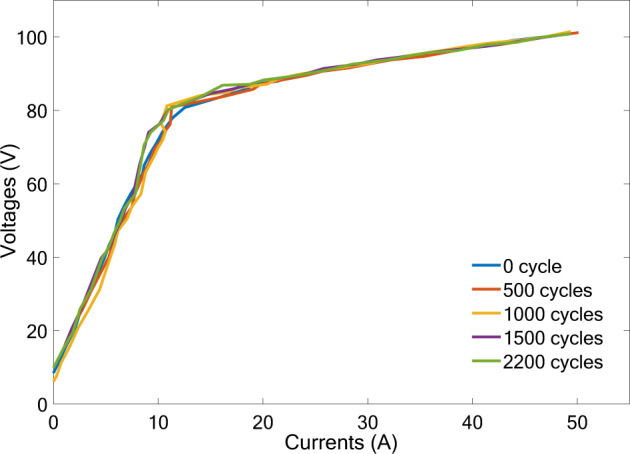
Accelerated degradation test of the commercial AWE under the control of MMSOEC strategy. The adopted accelerated degradation test protocol is that for one cycle, the input power is changed according to the sequence of 10%, 20%, 30%, 50%, 70%, and 100% of rated electrical power at 60 °C and every operation state lasts 1 min, namely one cycle lasts 6 min. During the whole accelerated degradation test, the temperature of the electrolyzer is maintained at 60 °C, and 2200 cycles are conducted in total. After certain cycles, the voltage-current curves of the commercial AWE are re-measured from 0 to 100 V. The experiment was repeated five times. The source data for Fig. 9 are available in Dataset 1.

Fig. 9 shows the influence of the proposed MMSOEC strategy on the lifetime of the commercial AWE. Through the results of the accelerated degradation test, it can be seen that the MMSOEC strategy has no obvious influence on the voltage-current curves of the AWE. That is, the lifetime of the AWE has no obvious degradation under the control of the MMSOEC strategy. Because for the MMSOEC strategy, the off time of the pulse current in the low-load state is very short (<100 ms), there is not enough time to form the reverse current, which usually needs the off time lasts several minutes^19–21^. During the off time of the pulse current under the MMSOEC power supply, the discharge current of electrical double-layer capacitors is dominated, which is non-Faradic. Hence, the MMSOEC strategy does not affect the durability of electrodes and does not influence the lifetime of the AWE.

## Discussion

We have demonstrated the inefficiency and inconsistency problems of low-load AWEs, which greatly limit the operation range of AWEs driven by RESs. It is shown that the operation process of a single cell cannot be simply generalized to the whole electrolyzer; more is different. Through the detailed operation process analysis of AWEs and the established equivalent electrical model, the inefficiency and inconsistency mechanisms are revealed. It is found that the shared electrolyte channel and relatively larger equivalent low-load resistance are the main reasons. That is, the physical structures and electrical characteristics but not the chemical properties of AWEs have a decisive influence on the low-load performance. Based on this, a multi-mode self-optimization electrolysis converting strategy is proposed to enhance the efficiency and consistency of low-load AWEs. Its effectiveness is verified not only by a lab-scale test system but also by a 2 Nm^3^/h commercial AWE. Especially compared to the conventional dc power supply, the maximum efficiency improvement can exceed two times, and the operation range can be extended to 10% of the rated load. In this paper, just fixed frequency and pulse amplitude are adopted; if the pulse currents can be self-adaptively adjusted according to different operating conditions, the low-load performance of AWEs can be further improved. The proposed method just changes the power supply and does not need to modify the components of electrolyzers; it can be easily generalized and can facilitate hydrogen production from RESs.

## Methods

### Large-scale commercial AWE experiment platform

**Fig. 10:**
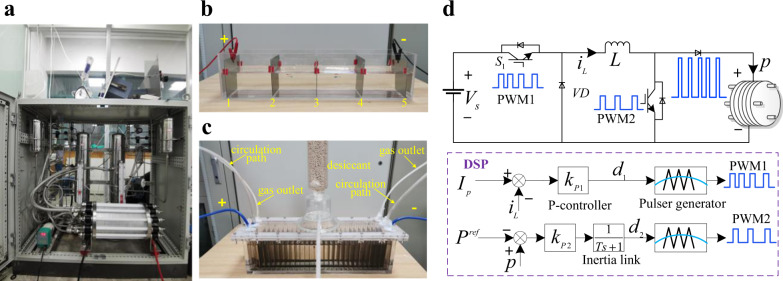
Experiment platform. **a** Commercial AWE (10 kW). **b** Simple AWEs for the observation. **c** Lab-scale test electrolyzer (3 kW) for the results verification. **d** Structure of the multi-mode self-optimization electrolysis converter and its control method in the low-load state.

As shown in Fig. 10a, the 2 Nm^3^/h commercial AWE is from Suzhou Jing Li Hydrogen Equipment Co., Ltd. This commercial AWE is equipped with proton flowmeters (RHE26, Rheonik Co., Ltd.), dehumidifiers and dew-point hygrometer (SD-P, Alpha Moisture Systems), analyzers of H_2_ content in O_2_ exhaust (NFY-3A, Xi’an Tiger Electric Technology Co., Ltd.), analyzers of O_2_ content in H_2_ exhaust (NFY-2A, Xi’an Tiger Electric Technology Co., Ltd.) and so on. The exhaust is about one bar. The used electrolyte is an aqueous solution of KOH (Type I, Huarong Chemical Co., Ltd.) at 30 wt.%; this solution is stirred for at least 10 min and is mixed thoroughly. Through dehumidification and purification, the purity of the generated hydrogen is larger than 99.999%. Therefore, it can measure the system efficiency accurately for large-scale hydrogen production.

### Small-scale simplified AWE experiment platform

As shown in Fig. 10b, the simple self-made AWEs mainly consist of two parts, the flumes and bipolar plates. The flumes are made of acrylic boards (PMMA, Shimao Co., Ltd.), which are transparent and convenient for observation. The thickness of acrylic boards is 5 mm. The bipolar plates are 304 stainless steel (304, Zhongzhiyuan Stainless Steel Products Co., Ltd.), which contains 18% Cr and 8% Ni. These bipolar plates are of different thicknesses for various experiments, as shown in Figs. 3,  5 and  6. Before using these plates, they are washed with the aqueous solution of KOH with high concentration to clear the surface oil contaminants. Then, they are immersed in a clean aqueous solution of KOH for 30 min. Similar to the commercial AWE, the used electrolyte is an aqueous solution of KOH (Type I, Huarong Chemical Co., Ltd.) at 30 wt.%; this solution is stirred for at least 10 min and is mixed thoroughly. The operating states of different cells are monitored by the underwater camera (CT60, Shenzhen Avanline Digital-tech Co., Ltd.), which can capture the gas adhesion on plates. All used materials and devices are commercially available.

Fig. 10c shows the lab-scale test electrolyzer used for the verification of the proposed MMSOEC strategy. Compared to Fig. 10b, the size is bigger, and the number of cells is larger. In addition, there is a seal cover with several gas outlets over the flume to collect gases. Behind the gas outlets, there are desiccant links to clear the water of gases. In this way, the system efficiency can be calculated by measuring the produced gases. At the same time, there are circulation paths to circulate the electrolyte and maintain its concentration.

### Multi-mode self-optimization electrolysis converting strategy

The multi-mode self-optimization electrolysis converter circuit is shown in Fig. 10d. The input voltage ${V}_{s}$ is 180 V (PSI 91500-30 3U, Elektro-Automatik Group). For the low-load state, ${V}_{s}$ is regulated by a power semiconductor switch ${S}_{1}$ to make the inductive current ${i}_{L}$ to be maintained at ${I}_{p}$ through a proportional controller. Switch ${S}_{2}$ is on and off alternatively; its duty ratio ${d}_{2}$ is regulated by a proportional controller with a first-order inertial link according to the desired system power or hydrogen production rate. Then, ${d}_{2}$ is sent to a pulse generator to form PWM waves. These PWM waves regulate the switch ${S}_{2}$ in real time, and desired pulse current is generated finally. For the high-load state, switch ${S}_{2}$ keeps the off state, the inductive current ${i}_{L}$ is not fixed and is regulated according to the desired system power or hydrogen production rate. The whole control algorithm is realized in a DSP microcontroller.

The filter inductor *L* is used to smooth the pulses generated switch ${S}_{1}$ and to form the steady current. At the same time, a fly-wheel diode *VD* is also needed because of the introduction of *L*. The key parameters of the adopted multi-mode self-optimization electrolysis converter are shown in Table 1.

**Table 1:** Parameters of the multi-mode self-optimization electrolysis converter.

Parameters	Value/type
${V}_{s}$(input voltage)	180 V
$L$(filter inductance)	2 mH
${S}_{1}$ switching frequency	10 kHz
${S}_{2}$ switching frequency	10 Hz
Microcontroller	Texas Instruments TMS320F28335
Switch tubes module	Mitsubishi PM300CL1A060
Current control loop	${k}_{P1}=0.04$
Power control loop	${k}_{P2}=0.05\,$, $T=10$

### Electrical characterization

The commercial AWE (10 kW) and lab-scale test electrolyzer (3 kW) are powered by the multi-mode self-optimization electrolysis converter, while the simplified AWE is powered by a low-voltage source (KA3005D/P, Korad Technology Co., Ltd.). The terminal voltages and input currents of these AWEs are monitored by an oscilloscope (MSO-X 3104 A, Agilent Technologies), among which the voltages are measured directly, and the currents are measured through a high-precision hall sensor (ZQM150LTBS, Zhuqingkeji Co., Ltd.). By directly measuring the terminal voltages of AWEs, the influence of voltage drop caused by the wire can be avoided, especially for large-current situations.

### Efficiency calculation and measurement

The efficiency of AWEs is defined as Eq. (1). The HHV of hydrogen is chosen as 141.88 MJ/kg or 12.67 MJ/Nm^3^. For the commercial AWE, the hydrogen production is measured through the proton flowmeter (RHE26, Rheonik Co., Ltd.), which can measure the hydrogen mass (g). The input electrical energy is measured by the power analyzer (PA6000H, Guangzhou ZHIYUAN Electronics Co., Ltd.), which can measure the terminal voltage, input current, and electric energy consumption (kWh) of the electrolyzer.

In conclusion, the system efficiency for the commercial AWE is calculated as

4 $$\eta =\frac{39.41}{1000}\cdot \frac{M}{E}$$

where $M$ is the accumulated hydrogen mass (g), $E$ is the electric energy consumption (kWh).

For the lab-scale test electrolyzer, the total volume of the electrolytic hydrogen and oxygen mixture (H_2_:O_2_ = 2:1) is measured. Then, the hydrogen volume can be obtained. Based on the gas state equation, the hydrogen volume under the standard temperature and pressure can be further derived. It should be noted that the hydrogen and oxygen mixture is explosive; any open flame should be avoided.

In conclusion, the system efficiency for the lab-scale test electrolyzer is calculated as

5 $$\eta =\frac{3.52}{1000}\cdot \frac{V}{E}$$

where $V$ is the equivalent accumulated hydrogen volume (L) under the standard temperature and pressure, $E$ is the electric energy consumption (kWh).

### Supplementary information

Description of Additional Supplementary File

Supplementary Dataset 1

## Data Availability

The source data for Figs. 1b, c, 2b, 5b, 6b, 8a, b, and 9 are provided in Supplementary Dataset 1. The data that support the findings of this study are available from the corresponding author upon reasonable request.
