# Supplementary material for: Efficiency and consistency enhancement for alkaline electrolyzers driven by renewable energy sources
Source: Commun Eng. 2023 May 3;2:22. doi: 10.1038/s44172-023-00070-7 (PMC10956030; doi:10.1038/s44172-023-00070-7)
Supplement: Supplementary file 1 — Description of Additional Supplementary File [file 44172_2023_70_MOESM1_ESM.pdf]

Description of Additional Supplementary Files

File name: Supplementary Data 1

Description: Source data underlying the graphs presented in the main figures are included in an Excel file.
